# Successful Clearance of Persistent Methicillin-Resistant *Staphylococcus aureus* Bacteremia with Daptomycin, Linezolid, and Meropenem Salvage Therapy

**DOI:** 10.1155/2019/5623978

**Published:** 2019-06-10

**Authors:** Grant Shaddix, Kalindi Patel, Matthew Simmons, Kelsie Burner

**Affiliations:** ^1^WVU Medicine Berkeley Medical Center, Department of Pharmacy, 2500 Hospital Drive, Martinsburg, WV 25401, USA; ^2^WVU Medicine Infectious Diseases, Department of Infectious Diseases, 2010 Doctor Oates Drive, Suite 103, Martinsburg, WV 25401, USA; ^3^WVU School of Pharmacy, Robert C. Byrd Health Sciences Center, 64 Medical Center Drive, P.O. Box 9500, Morgantown, WV 26506, USA

## Abstract

*Staphylococcus aureus* is one of the most virulent Gram-positive organisms responsible for a multitude of infections, including bacteremia. Methicillin-resistant *Staphylococcus aureus* (MRSA) is of special concern in patients with bacteremia. Due to its associated poor clinical outcomes, morbidity, and mortality, the superlative salvage regimen for persistent MRSA bacteremia remains uncertain. An 85-year-old white female presented with persistent methicillin-resistant *Staphylococcus aureus* (MRSA) bacteremia. Empiric antibiotic therapy with linezolid was initiated prior to blood culture results. Once MRSA bacteremia was confirmed, alternative antibiotic therapy with daptomycin was initiated. Blood cultures remained positive for MRSA despite three days of daptomycin therapy after which ceftaroline was added to the antibiotic regimen. Blood cultures remained positive for MRSA despite seven days of combination therapy with daptomycin and ceftaroline. Salvage therapy was then initiated with daptomycin, linezolid, and meropenem. One day following initiation of salvage therapy, blood cultures revealed no bacterial growth for the remainder of the length of stay. This report supports the effectiveness of salvage therapy consisting of daptomycin, linezolid, and meropenem in patients with persistent MRSA bacteremia.

## 1. Introduction

Methicillin-resistant *Staphylococcus aureus* (MRSA) is concerning in patients with bacteremia and infective endocarditis, due to poor clinical outcomes associated with crude mortality rates as high as 22.3%, increased lengths of stay of 11.1 ± 10.7 days, and median total charges of $36,109 [1, 2]. The current Infectious Diseases Society of America (IDSA) clinical practice guidelines for the treatment of MRSA infections currently recommend vancomycin or daptomycin as the first-line treatment strategies for MRSA bacteremia; however, ambiguity in current practice raises specific challenges for treatment failure and persistent disease [1]. In addition to prompt antibiotic therapy, infectious foci identification, removal and/or drainage, and surgical debridement are the first-line treatment strategies for deep-seated MRSA infections [1, 3]. There is a lack of current literature providing evidence-based endorsement on continued treatment strategies. Persistent MRSA bacteremia is defined by the IDSA guidelines as positive blood cultures past seven days of adequate therapy at effective doses of appropriate antibiotics [1]. Guidance in situations of persistent MRSA bacteremia is currently directed solely through case-series and case reports involving daptomycin and vancomycin in combination with salvage antibiotics for clearance of blood cultures [4–10]. A patient is presented here with persistent MRSA bacteremia and clearance of blood cultures following escalation to salvage therapy with daptomycin, linezolid, and meropenem.

## 2. Case Summary

An 85-year-old white female presented to the Emergency Department with altered mental status, weakness, and shortness of breath. She was admitted with an initial diagnosis of acute hyponatremia, acute or chronic kidney disease, and leukocytosis of unknown etiology following a previous admission at another facility for an acute exacerbation of congestive heart failure with aggressive diuresis and subsequent discharge. Pertinent past medical history includes heart failure with preserved ejection fraction (EF = 56%) and chronic kidney disease stage-4. Vitals were stable upon admission, and initial clinical examination did not reveal abnormal findings. Baseline laboratory values revealed leukocytosis (21.3 × 10^3^/uL), elevated procalcitonin (0.78 ng/mL), serum creatinine (2.01 mg/dL), and severe hyponatremia (115 mmol/L). Blood cultures were obtained on admission with empiric antibiotics initially held. Empiric intravenous linezolid was then initiated following preliminary positive blood cultures with Gram-positive cocci in clusters with positive MRSA PCR. Three days later, blood cultures were confirmed positive for MRSA, infectious disease service was consulted, and antibiotics were modified from linezolid to daptomycin 6 mg/kg every 48 hours. Upon further exam, pressure ulcers on the right buttocks (stage 2) and coccyx (stage 1) were identified by wound care service. Various imaging tests were conducted to identify a source of infection with findings consistent with ileocolonic ileus requiring placement of a nasogastric tube by general surgery and colonic decompression colonoscopy by gastrointestinal service. One day following daptomycin initiation, C-reactive protein (188.3 mg/L) and procalcitonin (0.60 ng/mL) were elevated and transthoracic echocardiogram (TTE) was negative for endocarditis vegetation; however, blood cultures remained positive for MRSA. Three days following daptomycin initiation, blood cultures remained positive for MRSA; consequentially, intravenous ceftaroline 400 mg every 12 hours was added to the antibiotic regimen. Another TTE was conducted, which revealed eccentric calcification on the aortic valve but could not rule out vegetation with follow-up transesophageal echocardiography (TEE) revealing mild to moderate mitral and tricuspid regurgitation without vegetation. Four days following the addition of ceftaroline, renal function improved towards baseline. Positive blood cultures persisted prompting optimization of antibiotics to daptomycin 10 mg/kg every 24 hours and ceftaroline 600 mg twice daily. A final CT of the lumbar spine revealed findings indicative of discitis osteomyelitis at T6-7. Blood cultures remained positive for MRSA despite seven days of combination therapy with daptomycin and ceftaroline. Salvage therapy was initiated using daptomycin 10 mg/kg every 24 hours, linezolid 600 mg every 12 hours, and meropenem 1 g every 8 hours. One day following initiation of the salvage therapy, blood cultures revealed no bacterial growth for five consecutive days. Infectious disease service recommended de-escalation fourteen days following negative blood cultures to vancomycin for a total of forty-two days of therapy. The patient's Medical Power of Attorney then requested transfer to a long-term care facility under hospice care following inpatient treatment. Antibiotics were discontinued per family request seven days following negative blood cultures.

## 3. Discussion

Methacillin-resistant *Staphylococcus aureus* (MRSA) bacteremia is cited as having a mortality rate of up to 30% prompting aggressive treatment with appropriate intraveneous antibiotics [1]. Current IDSA guidelines cite that based on popular expert opinion, persistent bacteremia at or around day 7 of therapy should prompt reassessment to consider alternative therapy [1].

In the case illustrated above, blood cultures remained positive for MRSA despite 6 days of daptomycin at the standard dose of 6 mg/kg. Based on data published in 2017 by Timbrook et al. escalating daptomycin doses beyond 6 mg/kg provided more benefit in patients with a higher predicted probability of 30-day mortality (51% or greater) [4]. This continued deterioration of the patient's clinical status prompted the addition of antistaphylococcal therapy for urgent clearance of blood cultures. Ho et al. initially presented a case-series of six patients using ceftaroline salvage therapy in persistent MRSA bacteremia or endocarditis previously treated with vancomycin or daptomycin. In all six cases, the addition of ceftaroline salvage therapy resulted in rapid clearance of bacteremia shortly after initiation [5]. Sakoulas et al. demonstrated in another case-series report that the addition of ceftaroline to daptomycin may provide additional benefit in treating persistent staphylococcal bacteremia. Twenty-six cases of refractory staphylococcal bacteremia in ten medical centers found that the median time to *S. aureus* bacteremia resolution was approximately two days with daptomycin plus ceftaroline combination therapy. It was concluded that the combination provides synergy against MRSA through several proposed mechanisms and provided a viable option for persistent cases [6]. Similarly, ceftaroline has been administered in combination with vancomycin in Grisenko et al.'s study and as salvage monotherapy in cases compiled by Burnett et al. with success [7, 8]. When ceftaroline was used in combination with vancomycin in five cases of MRSA bacteremia, microbiological cure was reported in all patients, with one case demonstrating clearance after escalation beyond labeled dosages to ceftaroline 600 mg administered every eight hours [7]. As a monotherapy for salvage, clinical success was reported with an average clearance of blood cultures between 1.5 and 3 days following initiation in four different case-series and reports [8].

Thirteen days into the admission, in light of multiple therapeutic alterations and the absence of an identifiable source, salvage therapy was initiated by the infectious disease team. Park et al. conducted a prospective randomized unblinded trial of ninety persistent MRSA bacteremia cases to be treated with either a glycopeptide-based regimen or linezolid-based salvage regimen, with or without a carbapenem [9]. Although duration of persistence in positive blood cultures was longer in the linezolid-based salvage group, results suggested a trend towards a more beneficial 30-day mortality rate but failed to reach statistical significance. Conversely, a study published by Jang et al. in 2009 studied thirty-five patients with persistent MRSA bacteremia and compared cases receiving salvage therapy of linezolid with or without a carbapenem versus a vancomycin and aminoglycoside or rifampin based regimen [10]. Early microbiological response within seventy-two hours was significantly higher in the linezolid-based regimen than the comparison group (75% vs. 17%, *P*=0.006) and touted an 88% salvage success rate (*P* < 0.001). Overall, the MRSA-related mortality rate was lower (13% vs. 53%, *P*=0.03) for patients treated with a linezolid-based regimen than for vancomycin-based regimens.

In our case, blood cultures remained positive for MRSA after a total of thirteen days of appropriate intravenous antibiotics and escalation of the regimen. The patient's bacteremia cleared one day following the initiation of salvage therapy consisting of daptomycin, linezolid, and meropenem.

## 4. Conclusion

An antibiotic regimen consisting of daptomycin, linezolid, and meropenem may be an effective salvage regimen in treating patients with persistent MRSA bacteremia.
